# The Prominent Role of HMGA Proteins in the Early Management of Gastrointestinal Cancers

**DOI:** 10.1155/2019/2059516

**Published:** 2019-10-13

**Authors:** Nathalia Meireles Da Costa, Luis Felipe Ribeiro Pinto, Luiz Eurico Nasciutti, Antonio Palumbo Jr.

**Affiliations:** ^1^Programa de Carcinogênese Molecular, Instituto Nacional de Câncer José de Alencar Gomes da Silva, Rio de Janeiro, RJ, Brazil; ^2^Laboratório de Interações Celulares, Instituto de Ciências Biomédicas, Universidade Federal do Rio de Janeiro, Rio de Janeiro, RJ, Brazil

## Abstract

GI tumors represent a heterogeneous group of neoplasms concerning their natural history and molecular alterations harbored. Nevertheless, these tumors share very high incidence and mortality rates worldwide and patients' poor prognosis. Therefore, the identification of specific biomarkers could increase the development of personalized medicine, in order to improve GI cancer management. In this sense, HMGA family members (HMGA1 and HMGA2) comprise an important group of genes involved in the genesis and progression of malignant tumors. Additionally, it has also been reported that HMGA1 and HMGA2 display an important role in the detection and progression of GI tumors. In this way, HMGA family members could be used as reliable biomarkers able to efficiently track not only the tumor *per se* but also the main risk conditions related with their development of GI cancers in the future. Finally, it shall be a promising option to revert the current scenario, once HMGA genes and proteins could represent a convergence point in the complex landscape of GI tumors.

## 1. Introduction

Cancer represents one of the most challenging diseases since the last century, and the exponential growing in the knowledge of its molecular basis shall represent a singular opportunity to translate this knowledge in practical tools, able to effectively impact on life quality of the people affected by this malignancy. This hope mainly resides on the potential application of the recent cellular and molecular discoveries in oncology field, into better strategies for disease prevention, early detection, prognosis increment, and new therapeutic approaches [1]. In fact, the identification of cancer-specific biomarkers has revolutionized this disease management, by increasing the development of personalized medicine, besides changing the “deadly” paradigm commonly associated with cancer [2]. In this sense, HMGA family members (HMGA1 and HMGA2) comprise an important group of genes involved in cancer genesis and progression [3]. Additionally, it has also been reported that HMGA1 and HMGA2 possess an important biomarker potential for the detection and progression of gastrointestinal (GI) tumors [4–8]. GI tumors present a very distinct biology that reflects a myriad of differences in the etiopathology of these malignances, which includes embryonic origin, tissue architecture, and tissue renewal pattern, as well as different etiological factors [9]. In addition, a relevant heterogeneity is observed among GI cancers, concerning clinical aspects, such as the differences harbored in their detection and development [10–12]. For instance, the well-delineated molecular and histopathologic panorama observed for colorectal cancer development, from the premalignant lesion to invasive carcinomas, is not shared by esophageal and gastric tumors. On the other hand, the late diagnosis and high lethality rates observed for esophageal and gastric tumors do not characterize colorectal tumors [9]. Therefore, the search for reliable approaches able to efficiently track not only the tumor *per se* but also the main risk conditions related with their development could represent a great improvement for the management of GI cancers in the future.

## 2. HMGA Proteins

The high mobility group A (HMGA) comprises a protein family of small nonhistone chromatin factors involved in the regulation of gene transcription, acting through either enhancement or suppression of the activity of transcription factors, by remodeling the chromatin structure and orchestrating the recruitment of multiprotein complexes of transcription factors [13]. The HMGA protein family is composed by four members, which are encoded by two genes, *HMGA1* and *HMGA2*. *HMGA1* generates three transcript variants—*HMGA1a*, *HMGA1b*, and *HMGA1c*—whereas *HMGA2* encodes only one transcript—*HMGA2* [13]. HMGA family members are characterized by the repetition of three amino acid sequences, called “AT hooks” motifs, which bind preferentially to the minor groove of AT-rich sequences in the DNA [14]. Despite the fact that HMGA proteins do not behave as classical transcription factors due to the absence of an intrinsic transcriptional activity, HMGA1 and HMGA2 are capable of modulating the transcription of target genes by inducing alterations in the chromatin structure [15]. Concerning HMGA gene and protein expression patterns, they are significantly expressed during embryogenesis, whereas their levels are almost undetectable in adult tissues. Nonetheless, HMGA levels are frequently upregulated in several different neoplasms, being their overexpression associated with tumor poor prognosis [16]. In this sense, a comprehensive meta-analysis has recently reported the significant impact of high levels of HMGA2 mRNA and/or protein levels on the diminution of cancer patients' overall survival, e.g., of patients affected by renal cell carcinoma, head and neck cancer, hepatocellular carcinoma, and pancreatic ductal adenocarcinoma [17]. HMGA proteins play an important role in cell transformation mainly due to their ability in controlling the expression of genes involved in cell proliferation and invasion control [16]. Contrary to the well-identified upregulation of HMGA in neoplasms and its role in tumorigenesis, the mechanisms underlying their mRNA and protein level expression are not yet completely understood. Lately, it has been reported that epigenetic mechanisms may play an important role in this process. Specifically, long noncoding RNAs and miRNAs have been demonstrated to regulate both HMGA1 and HMGA2 expression. Additionally, two HMGA pseudogenes have been recently identified and described as capable of modulating HMGA protein levels since they act as a decoy, hampering their degradation by miRNAs [18, 19]. The involvement of HMGA1 and HMGA2 in tumorigenesis has been largely reported along the last years, once the aberrant expression of these genes possesses implications not only in the tumor biology but also in cancer management, characterizing *HMGA* genes as potential diagnosis and prognosis biomarkers for several different tumors [13].

## 3. HMGA and Esophageal Cancer

In the last years, the growing knowledge in cancer biology promoted the development of new tools for early detection and more specific treatment of most cancer types. The technological revolution, mainly represented by large-scale gene expression analysis, and the development of selective target drugs have been figuring as new and promising strategies in the management of the disease [20]. On the other hand, esophageal cancer (EC) remains poorly impacted by these new approaches, a fact that could be partially explained due to the relative unexploited biology of this tumor [11]. In this sense, despite the scientific evolution experienced during the last years, EC is still characterized as a highly lethal tumor that presents a disappointing scenario where the late detection and poor prognosis are associated with no increment in the therapeutic strategies available for this cancer type [21]. In this way, the identification of new biological markers and the understanding of their role in EC development and progression comprise a fundamental step to a deeper knowledge of the disease. In addition, EC is mainly represented by two main histopathological types: esophageal squamous cell carcinoma (ESCC) and esophageal adenocarcinoma (EAC) [11]. These two EC subtypes largely differ in several aspects that include etiological factors, geographic location, and molecular alterations [22]. In this way, it is known that ESCC and EAC present alterations that are not necessarily shared and include EGFR amplification, differential patterns of DNA methylation, microRNA expression, and alterations in genes particularly involved in the regulation of the cell cycle [23]. On the contrary, it is well established that the most frequent alterations in both ESCC and EAC are the mutations in the tumor suppressor gene *TP53* (tumor protein p53) that is present in about 70–80% of EC cases [24]. Therefore, the improvement in EC molecular landscape knowledge is a mandatory step to upgrade the management of patients affected by this tumor. Moreover, this notion is particularly relevant along the evolution of EAC, once this histotype displays a relatively consolidated natural history, being the influence of obesity, gastroesophageal reflux disease (GERD), and Barrett's esophagus (BE) well-known risk factors associated with EAC genesis and progression [23]. BE is a metaplasia characterized by the replacement of the squamous epithelium by a columnar intestinal-like epithelium that has been associated with an increasing risk of EAC development [6]. In fact, the search for alterations in Barrett's metaplasia has been faced as the “lost link” along the development of EAC, since this condition often precedes the onset of the malignancy [25]. Accordingly, increased expression of HMGA1 along BE's progression has already been reported. Additionally, HMGA1 expression was positive in all BE cases that displayed high-grade dysplasia, whether its expression was barely detected in the BE patients' samples without dysplasia or with low-grade dysplasia [6]. Thus, HMGA1 expression pattern, represented by the increase in its expression along BE progression, has been suggested as an important approach for early EAC detection, through the screening of BE patients. Accordingly, some other biomarkers, such as *TP53*, *CDKN2A*, *CTNNB1*, *CDH1*, *GPX3*, and *NOX5*, among others have also been described to this end recently [26]. Additionally, converging with the importance of obesity as a crucial and independent risk factor for both BE and EAC development [23, 26], it is largely known that HMGA1 plays a crucial role in the adipogenesis [27]. Therefore, the investigation of HMGA1 expression in obese patients could reveal important insights about EAC evolution, since obesity has been reported as an independent risk factor for EAC development, even in the absence of GERD, that is the main inductor of the BE [28]. In accordance with this hypothesis, HMGA1 could be increasingly expressed along tumor evolution. Our group has already reported that HMGA1 is highly expressed in EAC, but not HMGA2 [29]. Interestingly, and on the opposite way, we observed that HMGA1 expression was almost absent in ESCC samples, but not HMGA2, which expression was markedly positive [29]. In accordance with the difference in HMGA gene and protein expression patterns depending on EC histotype, Toyozumi and colleagues showed a significative difference in HMGA1 expression between EAC and paired healthy esophageal tissue samples [30]. Nevertheless, in this study, the potential of HMGA1 expression as a diagnostic biomarker was not evaluated, as well as HMGA2 expression in the EAC samples investigated. Otherwise, these observations are quite interesting, particularly when seen under the prism of the major differences (etiological factors, geographic location, and molecular alterations) that characterize the two EC main histotypes. In fact, HMGA2 differential expression has been mainly, but not exclusively, associated with squamous tumors [31–33], being its expression related with several aspects of tumor evolution, particularly, invasion and metastasis [34]. As a matter of fact, during the progression of carcinomas, adhesion loss, extracellular matrix remodeling, and cytoskeleton reorganization promoted by epithelial mesenchyme transition (EMT) increase the malignant cell migration and invasion [35]. Besides consisting of a hallmark of tumor progression, EMT is also classically activated during healing process by several cytokines, particularly by transforming growth factor *β* (TGF-*β*) [36]. In an elegant study, Thualt and colleagues showed that canonical TGF-*β* signaling pathway activates SMAD proteins, ending up in the induction of the expression of transcription factors involved with EMT process [37]. In the same study, the authors demonstrated that EMT activated by TGF-*β* cannot occur in the absence of HMGA2, due to the fact that the activity of EMT transcription factors Twist, Slug, and specially Snail, was dependent on HMGA2 expression. Since then, several studies have reported that HMGA2 plays an essential role in EMT activation [38–41]. Moreover, the clinical relevance of HMGA2 expression levels has also been reported in a study which showed a significant association between its increased expression and occurrence of lymph node metastasis [42]. Contrarily, HMGA1 has been primarily involved in the genesis and evolution of adenocarcinomas, displaying a promising role not only as a diagnostic and prognostic biomarker but also by participating in the control of important malignant hallmarks, such as cell cycle, proliferation, and apoptosis, through the regulation of the expression of key genes (cyclin A1, Rb, p53, and Bax) involved in this process [43]. Finally, it was recently reported by our group that HMGA1, but not HMGA2, is a promising biomarker for endometrial adenocarcinoma development [44]. Furthermore, we demonstrated that HMGA2 displays an interesting potential in the detection of the larynx squamous carcinoma and HMGA1 expression does not seem to play any significant role in laryngeal carcinogenesis [33].

## 4. HMGA and Gastric Cancer

Gastric cancer (GC) is historically a high incidence cancer. Around 70 years ago, GC has been figured as the most frequent type of cancer. Its frequency has diminished along the years; nevertheless, it still ranks as the fifth most incidence tumor worldwide, among men and women [45]. The exact causes for the decrease in GC cases along the years have not been totally elucidated yet; however, an improvement in food maintenance practices probably played an important role in this process [46]. Currently, the incidence of the gastric cancer is particularly high in the Asian countries, especially in Japan. Additionally, similarly to EC, GC development is also strongly related to the exposition of the etiological factors associated with its genesis, particularly, the amount of salt present in the diet and *Helicobacter pylori* infection [47]. Moreover, GC presents a high mortality rate, being a very poor prognosis tumor and responsible for 782,685 deaths worldwide last year [48]. The intestinal and diffuse types comprise the main GC histopathological subtypes, being well established that the intestinal type is the most frequently detected subtype and mainly associated with *Helicobacter pylori* infection, while the diffuse subtype occurs predominantly in young and female individuals [47]. Recently, enhanced efforts allowed the identification of the molecular alterations harbored by gastric tumors and, further, made possible its classification into four different molecular subtypes, according to the main alterations present within the different tumors [49, 50]. The molecular GC classification may be valuably employed together with its histopathology. In this sense, some molecular alterations frequently found in GC occur almost exclusively in one of the subtypes: for instance, amplification of EGFR, ERBB-2, and MET and *TP53* mutations occur predominantly in the intestinal subtype, whereas CDH1 and RHOA mutations are mainly detected in the diffuse subtype [49, 50]. On the other hand, GC evolution has been classically associated with a multistep sequence of histopathological alterations, which include the development of gastritis, atrophic gastritis, and intraepithelial neoplasia [51]. In this respect, it was previously reported that the microRNA Let7 expression is progressively lost during GC development, since a downregulation of Let7 expression is observed in autoimmune gastritis patients, a condition that is strongly associated with the development of gastric mucosal atrophy and GC carcinogenesis. Furthermore, it was also observed that Let7 expression is decreased in patients infected with *Helicobacter pylori*, being its expression restored upon infection eradication [45]. As a matter of fact, Let7 microRNA expression deregulation has been related with the development of several cancer types, including GC [52]. Moreover, even though the mechanisms involved in HMGA member gene expression regulation have not yet been completely elucidated, it has been shown that HMGA2 expression could be directly regulated by the members of Let7 microRNA family [53]. Furthermore, Let7 has also been implicated in HMGA2 biology, once its *in vitro* overexpression is able to drastically reduce HMGA2 expression, which culminates in the abrogation of EMT, an important phenomenon occurring during carcinogenesis, in which HMGA2 is known to play a crucial role [38, 54]. Additionally, the axis Let7/HMGA2 seems to be important not only for the initiation of GC but also for its recurrence, since Takahashi and colleagues demonstrated that the remaining gastric mucosal areas following surgical resection, which are highly related to GC recurrence, display a significant reduction in Let7a expression concomitantly with an increase in the EMT transcription factor Snail [55]. Furthermore, the authors reported that Let7a inhibition in well-differentiated GC cell lines was able to increase HMGA2 and Snail expression, thus corroborating the key role of Let7a and HMGA2 in triggering EMT during GC carcinogenesis [55]. Moreover, diet is already known as an important etiological factor associated with GC development, being the intake of nitrate-enriched food associated with the generation of mutagenic/carcinogenic compounds, such as nitrosamines and nitrosamides [56]. In this sense, in a study in which the classic model of gastric carcinogenesis, by using *N*-ethyl-*N*-nitrosourea (ENU), was employed, a significant reduction in Let7b expression was observed after 15 days of ENU treatment [57], being the importance of this member of Let family in the regulation of HMGA2 expression, demonstrated in an *in vitro* model of lung cancer progression [58]. In addition, the relationship between HMGA2 and nitrosamine compounds might be even deeper, once HMGA2 was demonstrated to be a key player in DNA damage repair induced by *N*-methyl-*N*-nitrosourea (MNU) [59]. Thus, due to the close association of HMGA2 with the etiological conditions associated with GC development (*Helicobacter pylori* infection, mucosal metaplastic transformation, and nitrosamine exposure), one could state that HMGA2 plays a remarkable role in gastric carcinogenesis. However, HMGA2 overexpression could also be figured as a potential prognostic biomarker for GC progression, since the deregulation of its expression has been associated with several clinical pathological parameters which include vasculogenic mimicry during GC progression and disease recurrence [7, 60, 61]. In this sense, a recent meta-analysis reported a significant association between increased levels of *HMGA2* and later tumor stage, lymph node metastasis, vascular invasion, and diminished overall survival of gastric cancer patients, thus pointing out its potential role as a prognostic biomarker for gastric tumors [62]. Finally, oppositely to other GI tumors, HMGA family expression biomarker potential in GC seems to be restricted to HMGA2 due to the fact that only one study reported the HMGA1 overexpression in GC, otherwise, no association between HMGA1 and any clinical pathological parameter nor disease onset was observed [7].

## 5. HMGA and Colorectal Cancer

Colorectal cancer (CRC) has been figured as one of the most prevalent solid tumors, occupying the third position in incidence and the second in mortality, in both sexes worldwide [48]. Hopefully, probably due to the highest prevalence rates of this tumor occurring in the western population, particularly in developed countries, the management of the disease has been improved in the last years, compared with other tumors, such as esophageal cancer [63]. Moreover, the high incidence rates of CRC in western countries are mainly related to their lifestyle, which includes hypercaloric diet, high intake of red meat, and tobacco and alcohol consumption [64]. Additionally, intestine inflammatory pathologies, like Crohn's disease, and especially, ulcerative colitis, represent the major clinical conditions related to CRC development [64]. CRC presents a well-established natural history, not only regarding the main risk factors associated with its development but also in what concerns the main molecular alterations harbored by these tumors [65]. In this sense, genomic instability in CRC is represented by distinct sets of molecular alterations that allowed the molecular subclassification of these tumors into four groups [65]. Therefore, the chromosomal instability (CIN) group is the most representative one and responds for nearly 85% of all CRC cases, being particularly characterized by the presence of mutations in the tumor suppressors *TP53* and *APC* [66]. Furthermore, the remaining groups are related to other molecular events, such as microsatellite instability, methylation pattern, and DNA damage repair [67]. In addition, this well-defined scenario, which characterizes CRC, also reveals that among GI tumors, CRC is the one which possesses the best-characterized relationship with HMGA family members. In this way, it has been previously reported that HMGA1 is expressed low in healthy, nontumor colorectal mucosa, whereas its expression gradually increments along CRC evolution [4]. In this study, the authors observed that HMGA1 expression progressively increases from adenoma with mild atypia to adenoma with severe atypia, and, finally, to CRC, thus showing that the alterations in the HMGA1 expression pattern comprise an initial event along malignant evolution, thus indicating a strong potential of HMGA1 expression levels to be used in CRC early detection [4]. In this sense, it was demonstrated that HMGA1 overexpression was able to induce the emergence of polyps in a transgenic mouse model [68], thus reinforcing the relationship between HMGA1 expression deregulation and the early steps of CRC carcinogenesis, since polyps represent a precancerous lesion that, when surgically resected, prevents CRC development [68]. Additionally, by using the same murine model and proteomic approach, Williams and colleagues showed that HMGA1 overexpression was able to alter fatty acids biosynthesis and to decrease taurocholic acid, being these results also observed in CRC tissue [69]. Thus, these data seem very interesting, once the alterations in both pathways have been demonstrated to be related to the neoplastic transformation [70]. Ultimately, it was recently showed that CRC patients displayed high levels of HMGA1 protein in the blood, compared to healthy individuals, thus revealing a potential use of HMGA1 expression levels as a noninvasive CRC diagnostic biomarker [71]. In addition, it was observed that aberrant expression of *HMGA2* could also be efficiently detected in the blood of CRC patients, compared to healthy individuals [72]. However, the methods employed for HMGA1 and HMGA2 expression measurement in the blood of the individuals were completely distinct: to evaluate HMGA1 expression, a monoclonal antibody-based platform was used, while HMGA2 detection was performed by using cell-free circulating RNA approach. However, despite the differences regarding the technical principle of the two methods, both approaches could represent an improvement in the management of CRC patients in the future, particularly in its detection, since *HMGA2* is also overexpressed in CRC tissue, in addition to its association with patients' poor prognosis [73, 74]. Furthermore, Fan and colleagues detected a low expression of miR-543, an HMGA2 regulator microRNA, in CRC samples. Moreover, the authors also showed a downregulation of miR-543 in a mice model of colitis-associated colon cancer, which consists in a pathological condition associated with CRC development [75]. Therefore, as well demonstrated for HMGA1, it seems that the aberrant expression of HMGA2 might be involved in the early steps of CRC development. Additionally, as cited before, alcohol consumption is considered an important risk factor for CRC development [64]. In this sense, it was already reported that the injury induced by ethanol is able to drastically reduce the expression of several microRNAs belonging to Let7 family, whose members are known to downregulate HMGA2 expression [64]. Finally, obesity is considered one of the main conditions associated with CRC development. The increase in calorie consumption in developing countries has been associated with an exponentially growing number of cancer cases, including CRC [76]. Furthermore, it has been reported that HMGA1 and HMGA2 play a dual role in the adipogenesis, being the expression of these genes associated with fat tissue development [27, 77]. Therefore, it is known that HMGA1 expression impairs adipocyte differentiation, while HMGA2 expression induces adipocyte differentiation, being these effects due to a down- or upregulation exerted by HMGA1/HMGA2 on key genes involved in adipogenesis [27, 77]. In this way, HMGA1 and HMGA2 altered expression could also be envisaged as a biomarker panel to track CRC risk patients, since, under obesity condition, these genes exhibit an antagonistic expression pattern.

## 6. Conclusion

GI tumors represent a heterogeneous group of neoplasms concerning their natural history and molecular alterations harbored. Nevertheless, these tumors share very high incidence and mortality rates worldwide and patients' poor prognosis. Thus, additionally to patients' suffering, GI tumors heavily impact public health systems worldwide. Therefore, the increment in the molecular knowledge of these malignancies represents an unique opportunity to expand the portfolio of strategies for prevention, diagnosis, and treatment. Furthermore, it shall be the best option to revert the current dark scenario of GI tumors and, thus, represents a mandatory step to improve the management of GI cancers, being HMGA genes and proteins a convergence point in the complex landscape of GI tumors.
